# Prevalence and predictors of parental willingness to vaccinate daughters against human papillomavirus in Sub-Saharan Africa: a systematic review and meta-analysis

**DOI:** 10.3389/fpubh.2025.1486262

**Published:** 2025-07-01

**Authors:** Daniel Asmelash, Wondaya Fenta Zewdia, Gossa Fetene Abebe, Desalegn Girma, Abdu Hayder Mohammed, Abyot Asres

**Affiliations:** ^1^Department of Medical Laboratory Science, College of Medicine and Health Sciences, Mizan-Tepi University, Mizan-Aman, Ethiopia; ^2^Department of Statistics, College of Science, Bahir Dar University, Bahir Dar, Ethiopia; ^3^Department of Midwifery, College of Medicine and Health Sciences, Mizan-Tepi University, Mizan-Aman, Ethiopia; ^4^School of Public Health, College of Medicine and Health Sciences, Mizan-Tepi University, Mizan-Aman, Ethiopia

**Keywords:** HPV vaccination, parental willingness, cervical cancer, predictors, Sub-Saharan Africa

## Abstract

**Background:**

Human papillomavirus (HPV) infection is a major health burden, especially in developing countries. Primary prevention through HPV vaccination has demonstrated excellent efficacy in preventing r cervical cancer incidence. Parental willingness on behalf of their daughters plays a crucial part in deciding whether they should get an HPV vaccine or not, which determines the vaccine coverage. The purpose of this study was to determine the pooled prevalence and predictors of willingness to vaccinate their daughters against the HPV vaccine in Sub-Saharan Africa (SSA).

**Methods:**

A literature search of studies was conducted using multiple databases including the Web of Science, Cochrane Library, PubMed, Google Scholar, Gray Literature, Embase, and African Online Journal. The search included studies that were published between 2014 and 2024. The inclusion criteria included studies that examined parental willingness to vaccinate their daughters with HPV vaccine in SSA. Data were extracted using Excel and analyzed using Stata™ Version 11, and methodological quality was assessed using the Newcastle-Ottawa Scale modified for cross-sectional studies. The meta-analyses were performed using the random effects model. The protocol was registered with PROSPERO (CRD42024584292).

**Results:**

A total of 20 cross-sectional studies with 9,182 participants were included in the meta-analysis. The pooled prevalence of willingness to vaccinate daughters with the HPV vaccine was 73% (95% CI: 65–81%). Younger parents (AOR = 1.61, 95% CI: 1.08–2.39), educational status (AOR = 2.27, 95% CI: 1.66–3.12), higher income (AOR = 3.13, 95% CI: 1.96–5.02), good knowledge (AOR = 2.28, 95% CI: 1.59–3.27) and a positive attitude (AOR = 4.83, 95% CI: 2.51–9.30) toward HPV vaccine were significantly associated with parental willingness to vaccinate their daughters.

**Conclusion:**

The findings showed a significant proportion of parents remained unwilling to vaccinate their daughters against HPV in SSA. The study also found that parental willingness was significantly higher among parents with good knowledge about HPV and its vaccine, positive attitudes toward vaccination, younger age, higher educational status, and higher income levels. These findings underscore the critical role of parental willingness in shaping policies and initiatives aimed at increasing HPV vaccination rates and reduce the incidence of cervical cancer.

## Introduction

Human papillomavirus (HPV) infection is a major public health problem because of its association with the incidence of cervical cancer (1). Globally, there were an estimated 604,127 cervical cancer cases and 341,831 deaths in 2020 (1). Additionally, cervical cancer remains the fourth most common malignancy in women, with Sub-Saharan Africa (SSA) having the highest incidence and mortality rates, accounting for more than four-fifths of the global burden (2, 3).

There is still high morbidity and mortality associated with cervical cancer, even though there are vaccines against HPV types that cause most cancers and testing is done using HPV-based tests. HPV vaccination is a primary prevention strategy for cervical cancer, targeting HPV types 16 and 18, which cause 70% of cases (4, 5). HPV vaccine coverage shows significant disparities across regions and countries (6). In developed countries, the coverage rate is more than 50% for adolescent girls, which results in a significant decrease in vaccine-associated HPV illnesses (7). However, only 3% of adolescents have been vaccinated against HPV, and approximately 44% of women are screened for cervical cancer in low- and middle-income countries (8). HPV vaccine uptake in low- and middle-income countries is affected by several factors, including limited healthcare, economic hardships, cultural beliefs, and logistical problems in vaccine delivery (6). Because of the high prevalence of HPV infection and to provide optimal protection, the HPV vaccination is recommended for preadolescent girls (9).

The high level of parental influence on children, particularly maternal support, is crucial in the decision-making process leading to better vaccination coverage. Parents are primary stakeholders whose participation can have a significant impact on HPV vaccine uptake for their daughters (10, 11). Many factors, such as knowledge of HPV and its consequences, attitudes toward vaccine safety and efficacy, access to healthcare services, cultural norms, and trust in healthcare providers and authorities, can all affect parental decisions to vaccinate their daughters (12, 13).

While the argument for parental willingness is essential in daughters’ HPV vaccine uptake, current research on this matter in SSA remains fragmented and lacks comprehensive synthesis. Literature on parental willingness for HPV vaccination reveals significant variation across studies, which is based mainly on socioeconomic status, educational status, and healthcare services availability. To achieve this objective, we conducted this systematic review and meta-analysis to have a significant public health impact in SSA by designing and implementing interventions to improve HPV vaccination among adolescent girls and identify predictors of parental willingness for ultimately increasing coverage rates and preventing cervical cancer. Our study is an important input into the global target to decrease HPV-related diseases through targeted vaccination programs.

## Methods

### Identification of articles

We searched articles using Web of Science, Embase, Cochrane Library, PubMed, Google Scholar, Worldwide Science databases, gray literature, and African Online Journal. To prevent unnecessary duplication, we searched for previous systematic reviews and meta-analyses in the PROSPERO database^1^. We used the following MeSH terms: HPV vaccination, knowledge, willingness, acceptance, parents, sub-Saharan Africa, associated factors and determinants along with the names of specific countries in this region. The articles were managed using EndNote X20 software. To be reflective of current parental perspectives we limited our review to studies published in the last decade (2014–2021).

### Inclusion and exclusion criteria

The inclusion criteria consist of cross-sectional studies in SSA that report the prevalence of parental willingness to vaccinate and/or predictors and were published between June 2014 and 1 June 2024. However, those that did not report outcomes related to the prevalence of parental willingness to vaccinate their daughters and duplicate studies were excluded. Additionally, studies in languages other than English, and publications such as editorials, commentaries, case reports, case–control, cohort, case series, expert opinions, qualitative studies, and conference abstracts lacking detailed data were also omitted.

### Data extraction and quality assessment

Relevant articles were searched by six authors (DA, AH, WFZ, AA, GFA, and DG), and any discrepancies among these authors were resolved through discussion. Data extraction was carried out using Excel, and the collected data were subsequently transferred to Stata™ Version 11 for further analysis. The protocol was registered with the PROSPERO number.^2^

Quality assessment of the included studies was evaluated by all authors independently using the Modified Newcastle-Ottawa Scale for Cross-Sectional Studies (14). According to the quality assessment, 12 of the 20 articles showed a low risk of bias, while 8 demonstrated a moderate risk of bias. The analyses were examined for compliance with the PRISMA guidelines to ensure transparency and reproducibility of methods. Adherence to PRISMA guidelines for study selection, data extraction, and reporting of results, as well as any discrepancies in quality assessment, were resolved through discussion among the authors (Table 1).

**Table 1:** Study characteristics and quality assessment of studies included in the systematic review and meta-analysis of parental willingness to vaccinate daughters against HPV in Sub-Saharan Africa.

No	Author(s), year	Year	Study design	Sample size	Country	Parental willingness (%)	Quality score (Point)
1	BN Azuogu. et al., 2019 (40)	2019	Cross-sectional	290	Nigeria	89.1	7
2	Akinleye HW. et al., 2020 (37)	2020	Cross-sectional	301	Nigeria	79.2	6
3	Rabiu KA. et al., 2020 (16)	2020	Cross-sectional	318	Nigeria	35.5	7
4	Dereje N. et al., 2021 (17)	2021	Cross-sectional	422	Ethiopia	94.3	7
5	Destaw A. et al., 2021 (18)	2021	Cross-sectional	502	Ethiopia	79.5	8
6	Balogun FM. et al., 2022 (41)	2022	Cross-sectional	678	Nigeria	96.8	8
7	Kolek CO. et al., 2022 (12)	2022	Cross-sectional	195	Kenya	90	6
8	Mabeya H. et al., 2021 (42)	2021	Cross-sectional	300	Kenya	95	7
9	Aragaw GM. et al., 2023 (19)	2023	Cross-sectional	721	Ethiopia	79.1	8
10	Nechi AK. et al., 2023 (43)	2023	Cross-sectional	330	Cameroon	33.6	7
11	Ogochukwu TN. et al., 2017 (44)	2017	Cross-sectional	311	Nigeria	84.6	6
12	Mihretie GN. et al., 2022 (20)	2022	Cross-sectional	638	Ethiopia	44.8	8
13	Alene T. et al., 2020 (21)	2020	Cross-sectional	899	Ethiopia	81.3	9
14	Wassie M. et al., 2023 (22)	2023	Cross-sectional	386	Ethiopia	80.3	7
15	Ezeanochie MC. et al., 2014 (45)	2014	Cross-sectional	201	Nigeria	70	6
16	Larebo YM. et al., 2022 (23)	2022	Cross-sectional	530	Ethiopia	84.9	7
17	Morhason-Bello IO. et al., 2015 (15)	2015	Cross-sectional	1,002	Mali	88.6	9
18	Ifrah S. et al., 2023 (46)	2023	Cross-sectional	278	Kenya	30	6
19	Kidie S. et al., 2024 (24)	2024	Cross-sectional	410	Ethiopia	72.9	7
20	Adesina KT. et al., 2018 (33)	2018	Cross-sectional	470	Nigeria	44.9	8

### Statistical analysis

Statistical analysis for our systematic review and meta-analysis was conducted using Stata™ version 11 software. We utilized the I^2^ statistic to assess heterogeneity among the studies included, and to synthesize the pooled odds ratios (ORs), data were extracted from 2 × 2 tables. To identify potential sources of heterogeneity, we applied a univariate meta-regression model to explore the impact of study-level covariates such as publication year, sample size, and study quality on effect size. Given the expected variability, we employed random-effects models to account for both within-study and between-study heterogeneity. Differences in socioeconomic status, healthcare access, cultural beliefs, and local vaccination policies are all likely to cause variations in the results of a meta-analysis involving various populations and geographic locations. Both within-study and between-study variability are taken into account by a random-effects model.

The overall estimate of parental willingness to vaccinate their daughters against HPV and the odds ratio (OR) for the associated factors with the 95% confidence interval (CI) was illustrated using a forest plot. To assess the agreement among researchers who selected studies and extracted data, Cohen’s coefficient was applied. Sensitivity analyses were performed to evaluate the robustness of the pooled estimates by systematically removing specific studies and examining their impact on the overall results. Publication bias was visually inspected using funnel plots and was statistically analyzed using Egger’s regression test and Begg’s rank correlation test.

## Results

### Study selection

**Figure 1:**
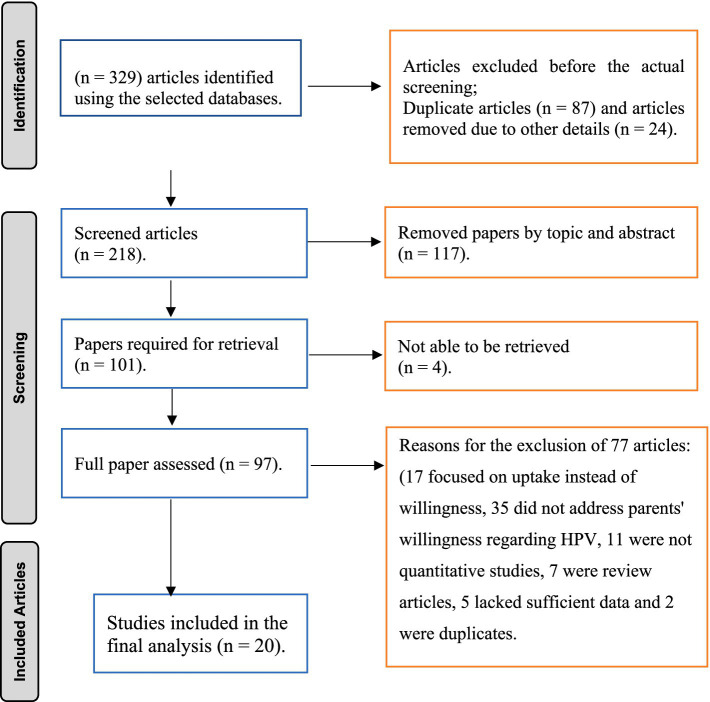
PRISMA flow diagram of study selection for the systematic review on parental willingness to vaccinate their daughter against HPV in Sub-Saharan Africa.

The literature search identified 329 studies, with all reviewers in complete agreement in selecting 97 relevant studies for full article assessment. Out of these, 20 met the inclusion criteria and the other 77 articles were excluded for several reasons: 17 focused on uptake instead of willingness, 35 did not address parents’ willingness regarding HPV vaccination for their daughters, 11 were not quantitative studies, 7 were review articles, 5 lacked sufficient data, and 2 were duplicates. In the end, 20 studies were included in the final meta-analysis (Figure 1).

### Characteristics of the articles

Twenty cross-sectional studies involving 9,182 participants were examined to assess the overall willingness of parents to vaccinate within communities in SSA. Most of these studies were carried out in West Africa, followed by East Africa, with sample sizes ranging from 195 (12) to 1,002 (15) participants. Among these studies, the highest willingness to vaccinate their daughters was reported in Nigeria (96.8%) (16), while the lowest was found in Kenya (30%) (2). Regarding study quality, the Newcastle–Ottawa Quality Assessment scale scores for all included studies varied from 6 to 9, indicating a good level of quality (Table 1).

### Data management and analysis

The overall prevalence of parental willingness to vaccinate their daughters was found to be 73% (95% CI: 65–81%). A random-effects model was employed to determine the prevalence of parental willingness due to the high heterogeneity among the studies (*I*^2^ = 99.2%). Additionally, high heterogeneity was also identified concerning factors influencing parental willingness, specifically knowledge (*I*^2^ = 86.7%) and attitude (*I*^2^ = 86.3%). In contrast, factors such as parental age (*I*^2^ = 6.1%), educational attainment (*I*^2^ = 20.4%), and income (*I*^2^ = 0%) did not exhibit significant heterogeneity.

**Figure 2:**
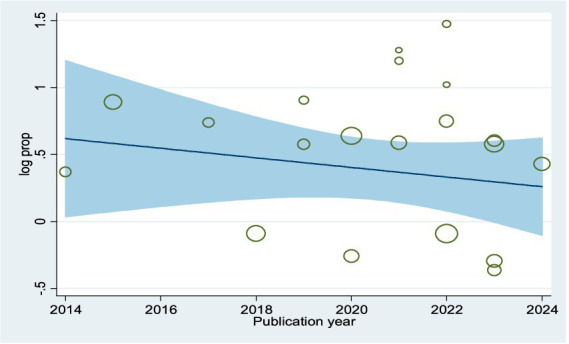
Meta-regression plot showing the trend of parental willingness to vaccinate their daughters against HPV in SSA, 2024, over 10 year’s period.

Visual assessment of the funnel plots, the results of Egger’s regression (*p* = 0.056), and Begg’s test (*p* = 0.098) were supported the conclusion of no publication bias (Supplementary Figure 1; Supplementary Table 1). The meta-regression trend analysis indicated that there was no significant change in the publication year from 2014 to 2024 concerning the pooled prevalence of parental willingness to vaccinate their daughters against HPV in SSA (*p* = 0.657) (see Figure 2). Additionally, findings from the sensitivity analysis showed that no individual study had a significant impact on the overall pooled prevalence of parents’ willingness to vaccinate their daughters in Ethiopia (Supplementary Figure 2).

### Level of parental willingness

**Figure 3:**
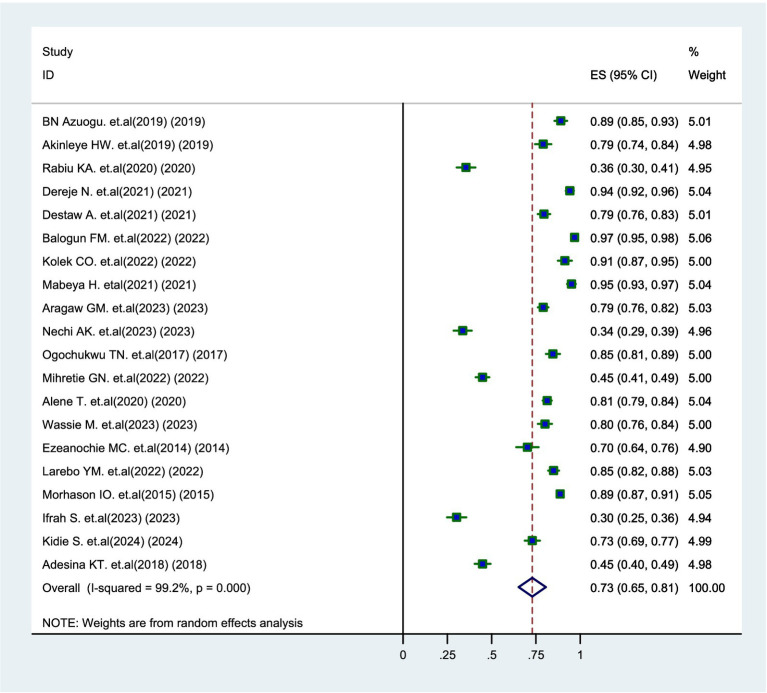
Forest plot of pooled prevalence of parental willingness to vaccinate daughters against HPV in SSA, 2024.

The overall prevalence of parents’ willingness to vaccinate their daughters was found to be 73% (95% CI: 65–81%), with significant variability observed across various studies (*I*^2^ = 99.2%, *p* = 0.000) (Figure 3).

### Association between knowledge level and parental willingness

**Figure 4:**
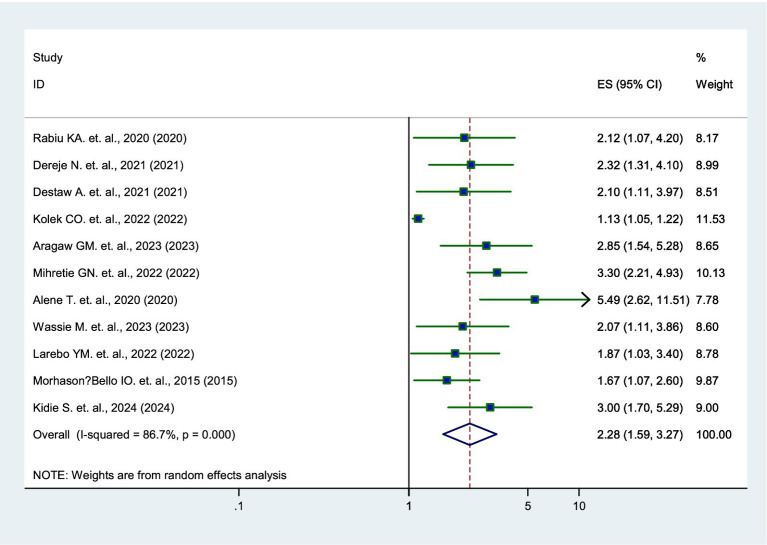
Forest plot showing the association between knowledge and parental willingness to vaccinate their daughters against HPV in SSA, 2024.

Eleven studies (12, 15–24) revealed that parents who had good knowledge about the HPV vaccine were 2.28 times more likely to vaccinate their daughters than those with poor knowledge (AOR = 2.28, 95% CI: 1.59–3.27) (Figure 4).

### Association between attitude and parental willingness

**Figure 5:**
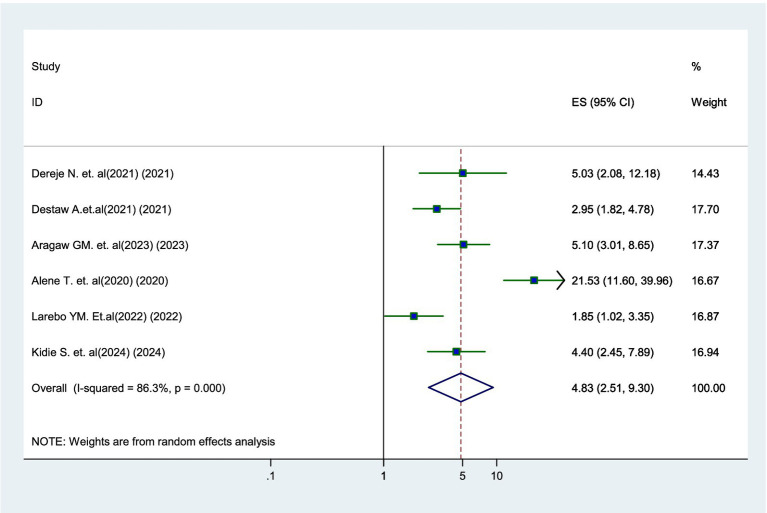
Forest plot showing the association between positive attitude and parental willingness to vaccinate their daughters against HPV in SSA, 2024.

Six studies (17–19, 21, 23, 24) identified a significant association between parents’ attitude and their willingness to vaccinate. Parents who had a positive attitude toward the HPV vaccine were 4.83 times more likely to have their daughters vaccinated than those with a negative attitude (AOR = 4.83, 95% CI: 2.51–9.30) (Figure 5).

### Association between parental age and willingness

**Figure 6:**
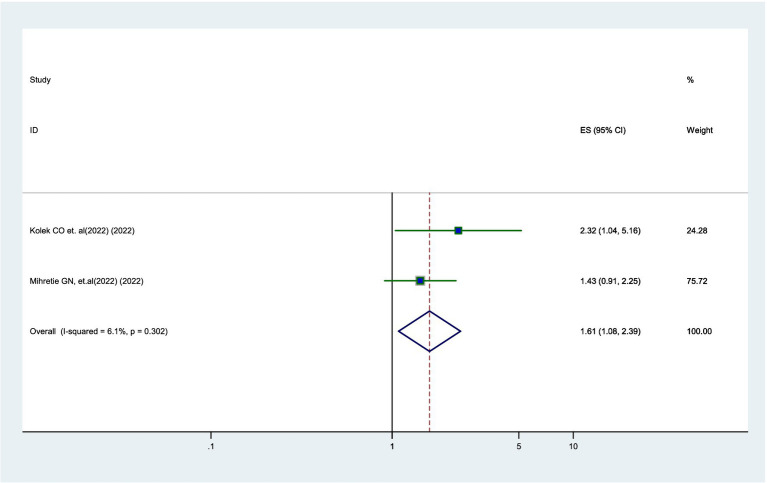
Forest plot showing the association between parental age and parental willingness to vaccinate their daughters against HPV in SSA, 2024.

According to two studies (12, 20), younger parents were 1.61 times more likely to vaccinate their daughters compared to older parents (AOR = 1.61, 95% CI: 1.08–2.39). Additionally, there was no significant heterogeneity observed among the studies (*I*^2^ = 6.1%) (Figure 6).

### Association between educational status and parental willingness

**Figure 7:**
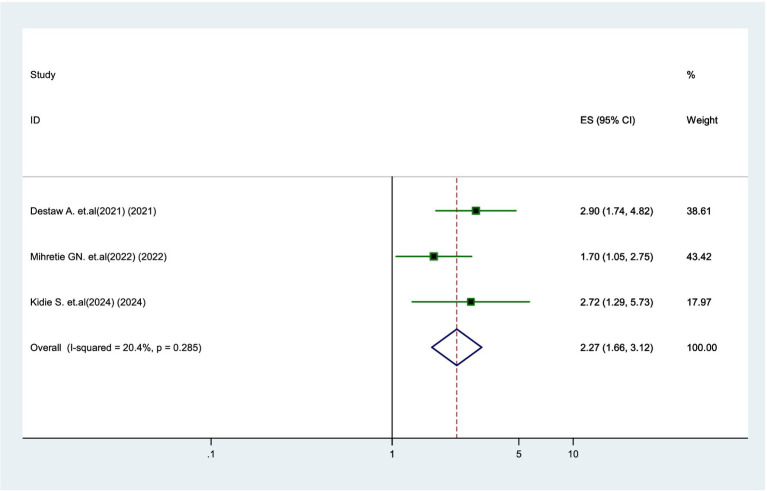
Forest plot showing the association between educational status and parental willingness to vaccinate their daughters against HPV in SSA, 2024.

Three studies (18, 20, 24) found that parents with primary education or higher were 2.27 times more likely to vaccinate their daughters compared to parents who were unable to read and write (AOR = 2.27, 95% CI: 1.66–3.12). There was no significant heterogeneity (*I*^2^ = 20.4%) among the studies (Figure 7).

### Association between income and parental willingness

**Figure 8:**
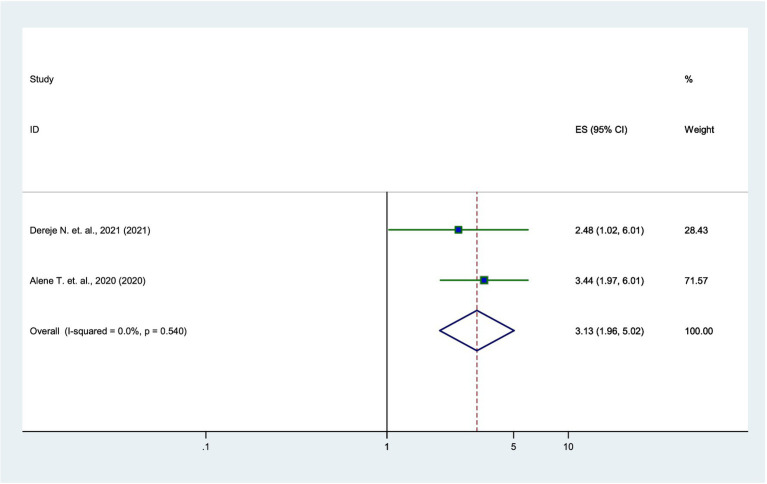
Forest plot showing the association between income and parental willingness to vaccinate their daughters against HPV in SSA, 2024.

According to the two studies (17, 21), parents with higher income were 3.13 times more likely to vaccinate their daughters than low-income parents (AOR = 3.13, 95% CI: 1.96–5.02), with very low heterogeneity (*I*^2^ = 0%) (Figure 8).

## Discussion

This study found two key findings about parental willingness to vaccinate their daughters for HV in Sub-Saharan Africa: First, a significant proportion of parents remained unwilling to vaccinate their daughters. Second, parental willingness was significantly higher among parents with greater HPV knowledge, positive attitudes, younger age, higher educational status, and higher income status.

The pooled prevalence of parental willingness to vaccinate their daughters was 73% (95% CI 65–81%). This figure aligned with studies conducted in Bozhou, China (71.5%) (25), Kolkata, Eastern India (74%) (26), and Shenzhen, China (73.9%) (27). In addition, this finding was significantly higher than those found in African Americans, USA (48%) (13), four provinces of China (46.39%) (28), Morocco (63%) (29), and Jinan, China (40.8%) (30). In contrast, it was lower than the figures reported in Poland (85.1%) (31), Qatar (77.9%) (26), and Mysore, India (79.1%) (32). The variation in willingness may be attributed to differences in sample sizes, socio-demographic characteristics, cultural and religious oppositions to HPV vaccination. Notably, the higher willingness reported in Poland may be associated with enhanced health literacy and better access to health information. This underscores the need for context-specific interventions that address regional barriers, such as improving healthcare infrastructure, increasing awareness, and addressing cultural resistance to vaccination.

Parents who were knowledgeable about the HPV vaccine were 2.28 times more likely to vaccinate their daughters compared to those with poor knowledge. This finding was supported by studies conducted in four provinces of China (28), Bozhou, China (25), and Shenzhen, China (27), highlighting the pivotal role of parental awareness in vaccine acceptance. Understanding the risks and complications of HPV and the advantages of its vaccination play an important role in parents’ acceptance of the vaccine, underscoring the need for targeted educational campaigns to address knowledge gaps (33, 34). This highlights the need for targeted educational campaigns to fix the knowledge gaps and dispel misunderstandings regarding HPV and the efficacy of the vaccines, particularly in underserved communities.

A positive attitude toward the HPV vaccine was a significant factor in parental willingness. This finding was consistent with findings from studies conducted in Black Americans (13), Mysore, India (32), Poland (31), England (35), and Indonesia (36). Parental willingness is shaped by their perceptions of the vaccine’s effectiveness, safety, and societal beliefs (37). Therefore, filling knowledge gaps through public health education initiative targeting parent with negative views about the HPV vaccine could improve vaccine acceptance.

Moreover, the current findings revealed that educated parents were twice as likely to vaccinate their daughters compared to illiterate parents. This finding was consistent with studies from Indonesia (38), four provinces of China (28) and Mysore, India (32). Additionally, higher income status was a significant predictor of parents’ willingness to vaccinate their daughters, which was consistent with findings in China (28) and among Arab Americans (39). The availability of health education and better financial resources contributes to their higher rates of vaccine acceptance. Therefore, policymakers should offer free HPV vaccine access to low-income families and integrate vaccination programs with current maternal and child health service networks.

The findings from this study underscore the importance of addressing regional disparities in parental willingness to vaccinate daughters against HPV in Sub-Saharan Africa. By tailoring public health interventions to meet the specific needs of communities and informing policy-making with robust data, stakeholders can work toward increasing vaccine uptake and ultimately reducing the burden of HPV-related diseases in the region. Through collaborative efforts and targeted strategies, we can foster a healthier future for girls across Sub-Saharan Africa.

Notably, parental age was found to be negatively associated with parental willingness to vaccinate their daughters, which was supported by similar finding in Mysore, India (32). This revealed that older parents may show greater resistance to modern health initiatives due to insufficient exposure to current health information and inherent misconceptions regarding vaccines. Therefore, targeted educational interventions that specifically address the concerns and misunderstandings of older parents could be instrumental to enhance vaccine acceptance.

## Conclusion

The pooled prevalence of parental willingness to vaccinate their daughters with the HPV vaccine was high. Furthermore, this study found several predictors associated with parental willingness to vaccinate their daughters, including age, knowledge, attitudes, educational background, and income levels. Targeted educational programs and equitable access to vaccination services are crucial for increasing vaccine coverage and will reduce the incidence of cervical cancer in SSA. There is a need for urgent re-prioritization of health programs in SSA toward improving research and training, screening, diagnosis, vaccination, data handling, and the overall management of the HPV vaccine in the region.

### Limitations

There are certain restrictions on this study. The results might not apply to all SSA nations, as some nations with few or no studies may not be well represented. The study’s generalizability to populations with limited access to screening and vaccination was constrained because it only included participants who had verified parental willingness to vaccinate their daughters. To improve the robustness and generalizability of results, future research should seek to overcome these limitations by using primary data sources, looking for unpublished studies, and making sure that the geographical representation is more balanced.

## Data Availability

The original contributions presented in the study are included in the article/Supplementary material, further inquiries can be directed to the corresponding author.

## Footnotes

^1^ http://www.library.ucsf.edu/

^2^ https://www.crd.york.ac.uk/PROSPERO/view/CRD42024584292
