# Supplementary material for: Prevalence and predictors of parental willingness to vaccinate daughters against human papillomavirus in Sub-Saharan Africa: a systematic review and meta-analysis
Source: Front Public Health. 2025 Jul 1;13:1486262. doi: 10.3389/fpubh.2025.1486262 (PMC12259698; doi:10.3389/fpubh.2025.1486262)
Supplement: Supplementary file 1 [file Data_Sheet_1.docx]

**Suppl. Fig 1.** Funnel plot of pooled prevalence of parental willingness to vaccinate their daughters against HPV in the Sub-Saharan Africa, 2024.


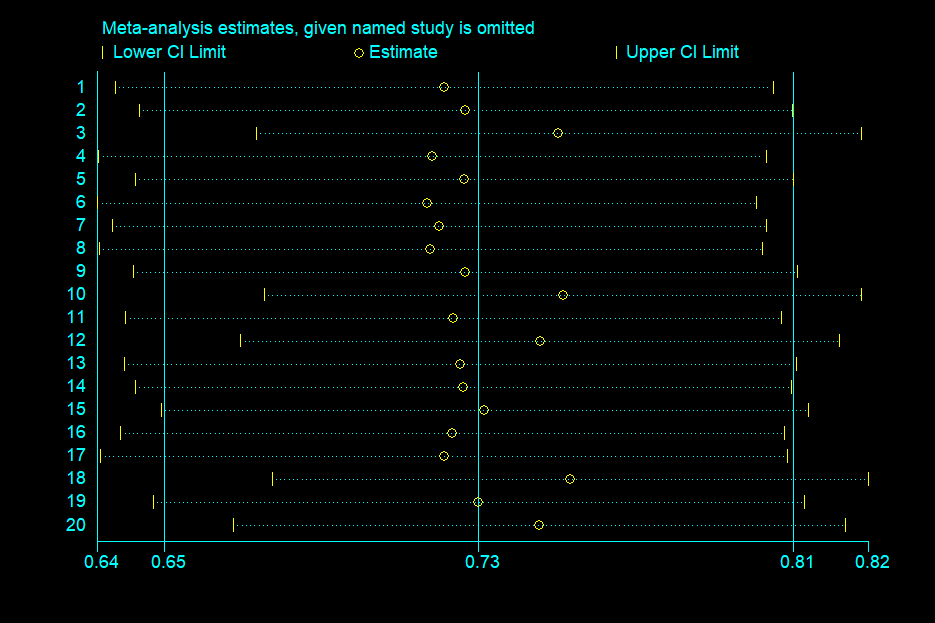


**Suppl. Fig 2.** Sensitivity analysis of pooled prevalence of parental willingness to vaccinate their daughters against HPV in the Sub-Saharan Africa, 2024.

**Suppl.Table 1.** Egger's Test for Assessing Publication Bias

| Std_Eff | Coef. | Std. Err. | t | P>t | [95% Conf. Interval] |
| --- | --- | --- | --- | --- | --- |
| slope | -.2514759 | -.2514759 | -0.79 | 0.440 | -.9208219 .41787 |
| bias | 5.514554 | 2.631192 | 2.10 | 0.056 | -.0133759 11.04248 |
